# Cognitive control of conscious error awareness: error awareness and error positivity (Pe) amplitude in moderate-to-severe traumatic brain injury (TBI)

**DOI:** 10.3389/fnhum.2015.00397

**Published:** 2015-07-07

**Authors:** Dustin M. Logan, Kyle R. Hill, Michael J. Larson

**Affiliations:** ^1^Department of Psychology, Brigham Young UniversityProvo, UT, USA; ^2^Neuroscience Center, Brigham Young UniversityProvo, UT, USA

**Keywords:** traumatic brain injury, TBI, cognitive control, error awareness, post-error positivity, Pe, ERP, event-related potential

## Abstract

Poor awareness has been linked to worse recovery and rehabilitation outcomes following moderate-to-severe traumatic brain injury (M/S TBI). The error positivity (Pe) component of the event-related potential (ERP) is linked to error awareness and cognitive control. Participants included 37 neurologically healthy controls and 24 individuals with M/S TBI who completed a brief neuropsychological battery and the error awareness task (EAT), a modified Stroop go/no-go task that elicits aware and unaware errors. Analyses compared between-group no-go accuracy (including accuracy between the first and second halves of the task to measure attention and fatigue), error awareness performance, and Pe amplitude by level of awareness. The M/S TBI group decreased in accuracy and maintained error awareness over time; control participants improved both accuracy and error awareness during the course of the task. Pe amplitude was larger for aware than unaware errors for both groups; however, consistent with previous research on the Pe and TBI, there were no significant between-group differences for Pe amplitudes. Findings suggest possible attention difficulties and low improvement of performance over time may influence specific aspects of error awareness in M/S TBI.

## Introduction

Awareness of behaviors, emotions, and cognitions is often adversely affected following moderate-to-severe (M/S) TBI (Hart et al., 1998, 2009; Sherer et al., 1998; Lanham et al., 2000; Port et al., 2002; Sherer and Hart, 2003; Larson and Perlstein, 2009). Individuals with M/S TBI frequently show difficulty recognizing poor performance and inappropriate behaviors and how their thoughts and behaviors are connected to potential environmental problems (Dockree and Roberston, 2011). Examples include repeating mistakes, committing social *faux pas*, and forgetting everyday tasks such as locking doors (Dockree and Roberston, 2011).

A secondary aspect related to error awareness in those with M/S TBI that plays a role in error detection is that of sustained attention (McAvinue et al., 2005). Sustained attention is the ability to maintain mindful, conscious processing of repetitive, non-arousing stimuli whose qualities would otherwise lead to habituation and distraction over time (Robertson et al., 1997). Sustained attention tasks include those that require detection of targets that occur infrequently over a long period of time. Individuals with M/S TBI typically show impaired performance on such tasks, most likely related to difficulty in allocating sufficient attention resources on long or boring tasks (Wilkins et al., 1987; Whyte et al., 1995).

Individuals with M/S TBI also show reduced error awareness compared to controls (Hart et al., 1998, 2009; O’Keeffe et al., 2004). Indeed, when error awareness is tested, there is a reduction in sustained attention when comparing those with TBI to controls (McAvinue et al., 2005). Both attention and awareness have strong electrophysiological connections seen through event-related potentials (ERPs; O’Connell et al., 2007; Orr and Hester, 2012; Samyn et al., 2014). One particular ERP component that has been linked to error-awareness is the error positivity (Pe) (also termed post-error positivity).

The Pe is a positive deflection in the ERP occurring 200–400 ms following an error (Falkenstein et al., 2000; Overbeek et al., 2005). The Pe reflects a representation of conscious error awareness in that the amplitude of the waveform covaries with the degree of awareness of an error (Niuwenhuis et al., 2001; Endrass et al., 2007; O’Connell et al., 2007; Dockree and Roberston, 2011). Further evidence in support of the error awareness theory of the Pe comes from findings showing that the salience of error-inducing information is positively correlated with Pe amplitude (Leuthold and Sommer, 1999; Ridderinkhof et al., 2009). Recent studies suggest that Pe amplitude is sensitive to the salience of an error and that salience secondarily may trigger error awareness (O’Connell et al., 2007; Ullsperger et al., 2010; Endrass et al., 2012). Notably, amplitude of the Pe is also correlated with conscious awareness of day-to-day functioning and behaviors (Larson and Perlstein, 2009; Larson et al., 2009; Wessel et al., 2011).

In recent studies our group has shown that the Pe and post-error slowing were similar between individuals with M/S TBI and healthy controls (Larson et al., 2007; Larson and Perlstein, 2009)—notably, however, Pe amplitude was related to poor ‘awareness of deficits,’ as rated by the discrepancy between patient and caregiver reports of functioning and cognitive abilities (i.e., cognitive sequelae). Importantly, none of these previous studies have directly manipulated error awareness or sought to understand the role of sustained attention or fatigue. Thus, in the current study we sought to further delineate the relationship between conscious error awareness and M/S TBI through the use of behavioral and electrophysiological indicators of error awareness. Specifically, we aimed to assess behavioral (response time and accuracy) and electrophysiological (Pe) indices of error awareness in individuals with M/S TBI. We hypothesized that individuals with M/S TBI would have fewer aware errors than neurologically healthy controls, that these differences would be more pronounced with increased time on the task (i.e., more errors on the second half of the task than on the first half), and that there would be group differences between M/S TBI participants and non-TBI controls for Pe amplitudes on aware error trials. With regard to the hypothesis about Pe amplitude, we note previous research that showed no difference between M/S TBI participants and controls on Pe amplitude; however, we hypothesized there would be differences in Pe amplitude in this study due to the direct measurement of error awareness using the error awareness task (EAT; described below).

## Materials and Methods

### Participants

The local Institutional Review Board (IRB) approved all study procedures, and all participants provided written informed consent. Study enrollment included 24 M/S TBI participants between the ages of 18 and 56 years (30.3 SD 11.7), and 37 neurologically healthy control participants between the ages of 20 and 49 years (23.2 SD 5.5). The control group was younger than the M/S TBI group, *t*(1,59) = -2.78*, p* = 0.009. There was no difference in years of education between the groups, *t*(1,59) = -0.59*, p* = 0.56 (M/S TBI *M* = 14.8 SD 2.5; controls *M* = 14.5 SD 1.2). A *chi*-squared test indicated that there were no significant differences between the groups on gender distribution, χ^2^(1) = 0.32, *p* = 0.57. The M/S TBI group had 16 males and 8 females (66.7% male) and the control group consisted of 22 males and 15 females (59.5% males). No participants were currently in rehabilitation. Five M/S TBI participants were included in behavioral (i.e., error rate, response time) analyses, but excluded from ERP analysis due to insufficient numbers of either aware or unaware error trials (i.e., fewer than six useable trials) or inability to complete the task accurately. Any person with fewer than six errors in any trial category was excluded due to a lack of stability and reliability in the average component waveform when using fewer than six trials in adult participants (Olvet and Hajcak, 2009). This left a primary total sample size of 56 for EEG analysis and evaluation (control *n* = 37, TBI *n* = 19). The sample size used for behavioral analysis included all participants from the TBI group (*n* = 24) that completed the EAT and the 37 neurologically healthy controls.

Participants were all native-English speakers. All but two of the M/S TBI participants were right-handed (one of which was not included in the ERP analyses). However, we determined that inclusion of two left-handed individuals was acceptable due to the small percentage (∼25–30%) of left-handed individuals who demonstrate some level of hemispheric language and memory differences (Lezak et al., 2012). Exclusion criteria included history of learning disability, ADHD, psychotic or bipolar disorder, uncorrected vision, language comprehension difficulties, recent substance dependence or history of neurological impairment other than TBI (i.e., stroke, epilepsy). Healthy controls were excluded if they had any history of mental health diagnosis in addition to the previous exclusionary criteria. All participants were screened for and excluded if they had color blindness as determined using the Ishihara pseudo-isochromatic color plates (Clark, 1924).

Participants were recruited via flyers placed at the local universities, medical centers, TBI support groups, the Utah Brain Injury Alliance, local medical providers, and through compiled lists of previous research participants who expressed interest in further participation in research. Control participants were recruited from undergraduate psychology classes that offer extra credit for research participation and flyers posted throughout the local community. Community controls (rather than orthopedic controls) were included due to recent findings indicating that community controls have no distinct disadvantage when compared to orthopedic control groups sometimes used in M/S TBI research (Mathias et al., 2013). Participants received course credit or $35 for participation.

#### Assessing Injury Severity

The M/S TBI group consisted of participants with chronic TBI who had sustained a TBI between ∼6 months prior to participation and less than 10 years from study participation. TBI severity was determined using duration of loss of consciousness (LOC), duration of post-traumatic amnesia (PTA), and Glasgow Comma Scale (GCS) score (Teasdale and Jennett, 1974) obtained from medical records and structured interviews. Moderate TBI was defined as the lowest post-resuscitation GCS score in a range of 9–12, PTA between 1 and 7 days, and LOC of more than 30 min, but less than 6 h (Bond, 1986; Bigler, 1990; Lezak et al., 2012). Severe TBI was defined as a GCS score of less than 9, LOC of greater than 6 h, or PTA of more than 7 days (Bond, 1986; Bigler, 1990; Lezak et al., 2012).

Participants were asked to bring with them or provide copies of medical records and neuroimaging for review to determine level of severity. If participants did not have access to their medical records, a signed release was requested in order to obtain copies of the records from their health care provider(s) and comprehensive interviews were conducted with the participant and/or significant other/caregiver to further determine level of severity. We employed retrospective interviewing methods to minimize confusion between disorientation and PTA (Shores et al., 1986). Retrospective techniques, while not ideal, have been shown to be reliable and valid for determining injury severity based on PTA (McMillan et al., 1996; King et al., 1997).

**Table 1** contains a summary of the TBI group severity classification information. Of the 24 M/S TBI participants there were 13 classified as severe and 11 classified as moderate according to the criteria noted above (see **Table 1**). Classifications were determined for nine of the participants from medical records and the remaining 15 from structured clinical interviews (McMillan et al., 1996; King et al., 1997). There were GCS scores reported in three (GCS = 3, 7, and 14) of the nine participants with medical records. The participant with a GCS score of 14 was not seen until the second day following the injury, after which he/she was hospitalized for 10 days and remained in PTA for 4 days according to medical records (and, thus was not classified as mild).

**Table 1:** Description of TBI participant injury severity and verification.

Age	Sex	Etiology	LOC hours	PTA hours	Months post
18	M	Fall	>0.5	0.8	8
27	F	BFT	504	504	99
41	F	Bike	0.5	24	82
22	M	MVA	336	336	99
24	M	MVA	384	1440	54
30	M	Fall	1	96	150
21	M	Fall	0.1	72	6
26	F	MVA	96	240	72
31	M	MVA	1080	336	69
35	M	MVA	0.5	1	16
28	F	Fall	0.3	36	98
24	M	MVA	144	144	27
23	F	MVA	^∗^	40	66
19	M	Fall	1	1.5	19
23	F	MVA	0.1	2016	31
24	M	Bike	<0.1	336	26
56	M	MVA	0.5	72	35
45	F	Fall	336	336	120
52	M	Bike	0.9	120	8
26	M	Bike	0.3	32	60
45	M	BFT	672	1344	39
51	M	Bike	18	18	105
18	M	Fall	120	120	6
18	F	MVA	1080	5760	25
		
		Mean:	198.9	559.4	54.9
		SD:	330.1	1223.8	41.1

BFT, blunt force trauma; Bike, cycling accident; MVA, motor vehicle accident; *indicates the participant denied LOC, but had a Ranchos Los Amigos Score on medical records of three upon admission.

### Error awareness task

The EAT was originally developed by Hester et al. (2005, 2009), and was adapted and used in this research with his permission. The EAT consists of a practice condition and the main task. In the practice task there are four steps. During the first step, participants were shown color-word stimuli and were instructed to press “1” for each stimulus. In the second step participants were instructed to continue with the previous instructions, but also told that if a word is repeated twice in a row they were to withhold their response when the repeated word was displayed a second time (consecutively repeated word equals no-go stimulus). Participants were then instructed that if they made a mistake and pressed the “1” button when they should have withheld their response they needed to press “2” on the next trial in order to indicate awareness of the error. In the third step participants were instructed to continue to press “1” for each incongruent stimulus, however, if the word was written in the same color of font as the written word they do not press any key (congruent stimulus equals a no-go trial). The last step reminded participants that if they did press a key on either a congruent trial or a repetition trial they were to signal they made an error by pressing “2” on the subsequent trial regardless of the type of stimulus shown during the “awareness” trial. The first phase (steps one and two) of the practice consisted of 50 trials and the second phase (steps three and four) consisted of 100 trials to ensure adequate learning of each rule. If a participant did not meet a 75% criterion indicating mastery of each step in the practice they were allowed to repeat that portion of the practice up to two more times in order to meet rule mastery criteria.

**FIGURE 1:**
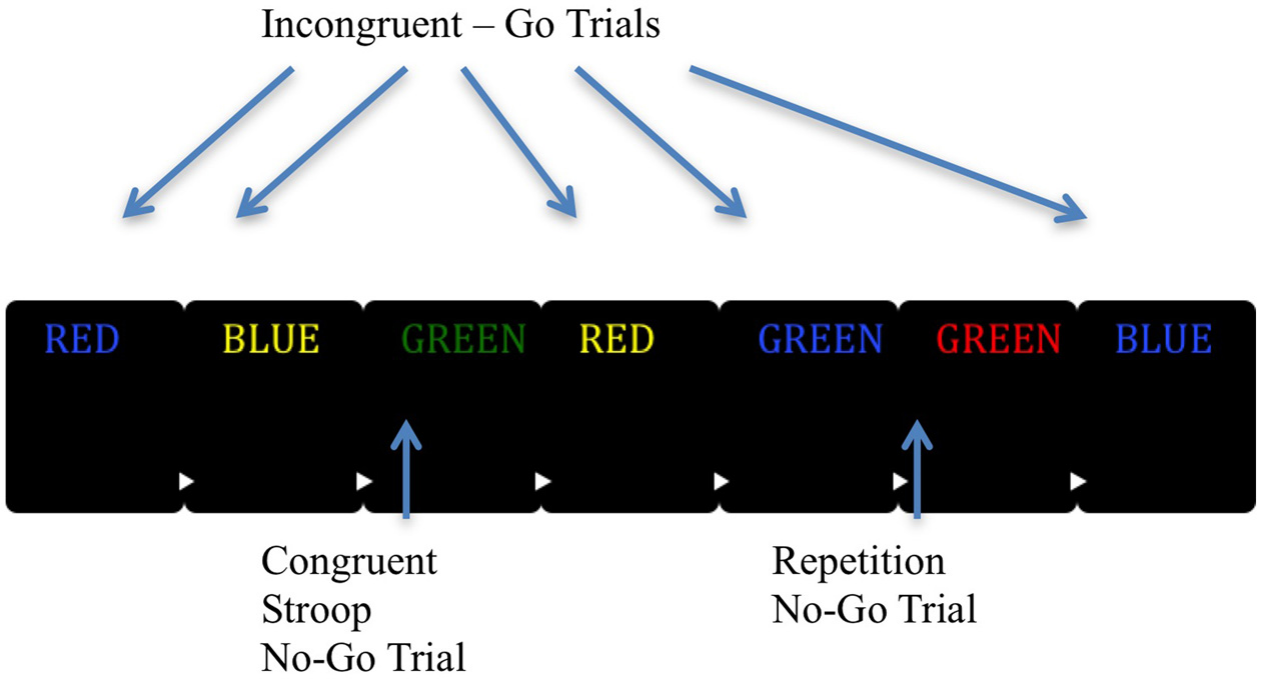
**Graphic representation of the EAT task.** The EAT presents a serial stream of single color words in incongruent fonts, with the word presented for 900 ms followed by a random inter-trial interval between 1000 and 1500 ms. Participants were trained to respond to each of the words with a single ‘Go trial’ button press, and withhold this response when either of two different circumstances arose. The first was if the same word was presented on two consecutive trials (Repeat No-go), and the second was if the word and color font of the word match (Congruent Stroop No-go). To indicate ‘error awareness’ participants were trained to press the error button on the trial following any commission errors. Adapted from “Neural Mechanisms Involved in Error Processing: A Comparison of Errors Made With and Without Awareness,” by Hester et al. (2005).

The main task is summarized in **Figure 1**. The task employed all of the rules the person was taught during the practice. They were to press “1” if presented with an incongruent stimulus and withhold their response if they saw a congruent stimulus or they saw a consecutively repeated word. If they did press a key when not indicated they were to signal that they made an error by pressing “2” on the next trial. Those no-go trials that were responded to and that were followed by a “2” button press were recorded as aware errors. Those trials where any response was provided on a no-go trial and was followed by a standard “1” button go response on the subsequent trial were recorded as unaware errors (Hester et al., 2005, 2009). Trials where participants made an initial go response on no-go trials, but then immediately responded with an awareness response before waiting to see the next trial were maintained as aware responses only if they then signaled awareness upon seeing the next trial. Trials only recorded the first participant response and were excluded if no response was recorded on go trials. Each word was presented for 900 ms with a random inter-trial interval (ITI) of between 1000 and 1500 ms. The task consisted of four blocks of 225 trials, including 46 no-go trials (23 incongruent and 23 repetitions) and 179 go trials per block for a total of 900 trials (717 go and 183 no-go).

### Electrophysiological Data Recording, Reduction, and Measurement

Electroencephalogram data was recorded from a geodesic sensor net with 128 scalp sites and Electrical Geodesics, Inc. (EGI; Eugene, OR, USA) amplifier system (20K gain, nominal bandpass = 0.10–100 Hz). Electrode placements enabled recording vertical and horizontal eye movements reflecting electro-oculographic (EOG) activity. Data from the EEG was referenced to the vertex electrode and digitized continuously at 250 Hz with a 24-bit analog-to-digital converter. A right posterior electrode approximately two inches behind the right mastoid served as common ground. Electrode impedance was maintained at or below 50 kΩ.

Electroencephalographam data was segmented off-line and single trial epochs rejected if voltages exceeded 100 μV, transitional (sample-to-sample) thresholds were greater than 100 μV, or eye-channel amplitudes were above 70 μV. For all participants, trials were considered bad and removed if more than 15% of channels were marked bad. Channels were marked bad if the fast average amplitude exceeded 100 μV or if the differential average amplitude exceeded 50 μV (e.g., Dien, 2010; Clayson and Larson, 2012). Data were digitally re-referenced to an average reference then digitally low-pass filtered at 30 Hz. Eye movement artifacts including blinks, saccades, and movements were corrected using independent component analysis as part of the open source ERP PCA Toolkit in Matlab (Dien, 2010).

For the Pe, event-related epochs were response-locked and extracted with a duration from 200 to 400 ms post-response from six centro-parietal sites surrounding Pz (54, 55, 61, 62 [Pz], 78, 79; see Clayson and Larson, 2012 for figure). The use of the mean amplitude procedure improves robustness to noise when compared to peak amplitudes, particularly in clinical groups (Clayson et al., 2013). Correct-response data for both components was collected using the same time window and electrodes.

### Neuropsychological Functioning and Mood Measures

Participants completed a short battery of measures aimed to characterize their current neuropsychological functioning and current mood. Measures included the Apathy Evaluation Scale – Self-Rating Form (AES; Marin, 1991), Beck Depression Inventory – Second edition (BDI-II; Beck et al., 1996), Wechsler test of Adult Reading (WTAR; Holdnack, 2001), and Repeatable Battery for the Assessment of Neuropsychological Status (RBANS; Randolph et al., 1998).

### Statistical Analyses

Statistical analyses were conducted using the statistical software package SPSS 21 (SPSS IBM, New York, NY, USA) and the ERP PCA Toolkit (Dien, 2010). No outliers were found using an outside boundary of two interquartile ranges of the median score for any observed variable. We examined demographic variables as a function of group to ensure groups were similar on age, education, number of trials for ERP analysis, and gender ratio using independent-samples *t*-tests and *chi*-square analysis, respectively. All accuracy and error awareness percentages were transformed using an arcsine transformation. We used the arcsine transformation because accuracy and error awareness percentages were derived from count data resulting in increased risk for binomial distributions and a significant negative skew. Due to significant skew the accuracy and error awareness percentages required the arcsine transformation to normalize the distribution.

Robust ANOVAs were calculated using the ERP PCA Toolkit to evaluate error awareness rates between M/S TBI and control groups. Robust ANOVAs were used in order to overcome the biasing effects of non-normality, (co)variance heterogeneity between groups, non-orthogonal groups, and to reduce Type I error (Keselman et al., 2003; Dien, 2010). The seed for the number generation was set at 1,000, and the number of iterations used for bootstrapping was 50,000 for all robust ANOVA analyses (Dien et al., 2006, 2010; Clayson and Larson, 2012). We decomposed significant interactions using Fisher’s least significant difference approach, controlling for family wise Type I error. We completed additional robust ANOVAs for no-go accuracy, accuracy rates by lure type (e.g., color and repeat), and response times for go, error, aware errors, unaware errors, and awareness response trials between groups.

To address potential differences in task performance over time related to impairments in attention or fatigue, we split the data from the EAT into an early half from trials 1–450 and a late half from trials 451–900. We then completed separate 2-Group × 2-Time (early, late) robust ANOVAs to compare groups on first and second half behavioral performance for each RT and accuracy condition. Unfortunately, there were insufficient trials (i.e., fewer than six per condition) in multiple categories for 13 out of the 19 M/S TBI participants leaving insufficient sample size to complete a full analysis. Early and late analyses of the ERPs were, therefore, not conducted and these analyses are included for the behavioral (i.e., response time [RT]/accuracy) data. To further assess group differences an accuracy percent change score was calculated for each behavioral condition by subtracting arcsine transformed early performance from late performance and then dividing by early performance ([Late – Early]/Early). Groups were then compared using independent samples *t*-tests. Zero order correlations were used to assess the relationship between indices of injury severity and EAT task performance.

## Results

There were significant between-group differences on levels of depression and apathy reported in the BDI-II, *t*(1,34.37) = -4.05*, p* < 0.001 and the AES, *t*(1,37.52) = -3.07, *p* = 0.004. The M/S TBI group reported higher levels of depression (*M* = 11.8, SD 7.8, range = 0–28) compared to controls (*M* = 4.5, SD 4.8, range = 0–21) and apathy (*M* = 30.3, SD 7.2, range = 20–50) compared to non-injured controls (*M* = 25.59, SD 5.05, range = 18–44). Notably, neither group’s mean depression scores met the threshold for the mild depression lower-bound score of 14 on the BDI-II. Similarly, neither group met criteria for elevated levels of apathy using the cut-score for elevated levels of apathy above 34 on the AES (Andersson et al., 1999). Thus, despite the group differences, levels of depression and apathy were not in a clinically significant range.

### Neuropsychological Performance

Analysis of neuropsychological data indicated that there were no significant differences between the M/S TBI and control groups on the RBANS Total score, *t*(1,32.01) = 0.36*, p* = 0.72, nor on the WTAR, *t*(1,59) = 0.09*, p* = 0.93, indicating no between-group differences on predicted pre-injury cognitive functioning or measured overall post-injury cognitive scores. No group differences for the RBANS subdomains of immediate and delayed memory, language, attention, and visuospatial processing (*t*s < 1.14, *p*s > 0.26; see **Table 2**) were found when comparing M/S TBI participants to healthy controls. The neuropsychological test findings suggest that the current sample of M/S TBI participants were functioning quite well cognitively despite their injuries. This is likely due to the time since their injuries or the single participant with M/S TBI that performed particularly well (see **Table 2**).

**Table 2:** Descriptive data of neuropsychological measures by group.

	M/S TBI (*n* = 24)			Control (*n* = 37)			
	Mean	SD	Range	Mean	SD	Range	*p*
WTAR (WAIS-III Predicted IQ)	109.3	9.5	82–121	110.5	7.4	89–120	0.92
RBANS total	95.8	22.3	54–145	97.6	12.2	75–122	0.72
Immediate memory	98.0	19.3	57–136	97.2	12.9	69–123	0.85
Visuospatial	104.3	13.6	75–126	102.6	14.7	62–126	0.66
Language	93.5	19.6	51–130	94.8	19.9	78–118	0.80
Attention	96.3	20.5	55–128	98.3	13.8	64–135	0.39
Delayed memory	92.6	21.3	48–131	98.1	12.8	56–125	0.26

*RBANS, Repeatable Battery for the Assessment of Neuropsychological Status; WTAR, Predicted Wechsler Adult Intelligence Scale -III Full Scale Intelligence Quotient score of the Wechsler Test of Adult Reading*.

### Behavioral Analyses for the EAT

#### Accuracy and Error Awareness

Accuracy and RT data for the M/S TBI participants and controls are included in **Table 3**. There were no significant differences between M/S TBI and control participants on percentage of aware errors, *T*_WJt_/c(1.0,37.2) = 0.89, *p* = 0.35. Similarly, there were no significant between-group differences for other measures of EAT accuracy including, no-go accuracy, *T*_WJt_/c(1.0,38.7) = 0.43, *p* = 0.51, and no-go accuracy broken down by lure type (i.e., repeat and color), *T*_WJt_/cs < 0.48, and *p*s > 0.49. Error awareness and error awareness separated by lure type (i.e., repeat and color) showed no significant group differences between M/S TBI and control participants, (*T*_WJt_/cs < 2.65, *p*s > 0.12).

**Table 3:** Behavioral data for M/S TBI and control groups on the error awareness task.

	M/S TBI (*n* = 24)		Control (*n* = 37)	
	Mean	SD	Mean	SD
No-go accuracy (% correct)	49	23	52	19
Repeat no-go accuracy	57	23	59	19
Color no-go accuracy	40	25	44	23
Error awareness (% of aware errors)	65	24	71	19
Repeat error awareness	59	27	70	19
Color error awareness	70	25	77	21
Unaware error proportion	35	24	27	16
Go RT (ms)	533.2	91.2	506.4	80.1
Error RT (ms)	543.9	96.5	536.8	101.5
Aware error RT	550.1	104.5	530.7	106.1
Unaware error RT	537.0	101.4	538.3	105.4
Error awareness RT (ms)	438.7	92.8	410.6	75.8

*Accuracy (percentage of correct responses) data presented in table are not arcsine transformed and represent the observed overall accuracy rate and percentages of errors a participant was aware of. P-values representing potential differences between groups are included within the body of the text*.

#### Response Times

There were no significant differences when comparing the M/S TBI and controls groups on RTs for overall performance on the EAT, *T*_WJt_/cs < 1.14, and *p*s > 0.29. A separate 2-Trial Type (Go, Error) by 2-Group robust ANOVA for RTs indicated a significant main effect of accuracy, *T*_WJt_/c(1,42.7) = 14.32, *p* = 0.001, with slower error- than go-trial RTs. There was no significant Trial Type × Group interaction when separated by lure type, *T*_WJt_/c(1,40.4) = 0.11, *p* = 0.73, or main effect of group, *T*_WJt_/c(1,42.7) = 1.45, *p* = 0.23 (see **Table 3**).

### Early-to-late Behavioral Performance

**Table 4** contains data comparing EAT first-half accuracy and RT performance with second-half accuracy and RT performance as a function of group. Overall, patterns of performance over time differed between the M/S TBI group and controls with the M/S TBI showing decreasing accuracy and maintained awareness over the course of the task. Control participants showed improved accuracy and awareness. Robust 2-Group × 2-Time (e.g., early, late) ANOVA for early and late EAT performance showed a significant main effect of time for awareness, *T*_WJt_/c(1,54.9) = 49.11, *p* < 0.001. There was also a significant main effect of group, *T*_WJt_/c(1,22.6) = 5.31, *p* = 0.03 and a Group × Time interaction, *T*_WJt_/c(1,54.9) = 53.34, *p* < 0.001. Decomposition of this interaction indicated that the control group had lower awareness early in the task, *T*_WJt_/c(1,29.4) = 35.55, *p* < 0.001, as well as an improvement in awareness from early to late, *T*_WJt_/c(1,35.0) = 77.56, *p* < 0.001. The M/S TBI group had similar awareness from early to late and similar to the control group at the later time point (*p*s > 0.12). A similar 2-Group × 2-Time robust ANOVA for accuracy produced a non-significant main effect of time, *T*_WJt_/c(1,36.1) = 3.71, *p* = 0.06. There was also a non-significant main effect of group, *T*_WJt_/c(1,34.8) = 0.29, *p* = 0.59 and Group × Time interaction, *T*_WJt_/c(1,36.1) = 0.45, *p* = 0.50. These patterns are presented below as a function of lure type as done by Hester et al. (2005, 2009) in their original analyses of the EAT^1^.

**Table 4:** Descriptive data and early to late behavioral performance change during the EAT as a function of group.

	M/S TBI (*n* = 24)					Control (*n* = 37)				
	Early		Late			Early		Late		
	Mean	SD	Mean	SD	*p*	Mean	SD	Mean	SD	*p*
No-go accuracy (% correct)	48	20	50	26	0.40	50	16	55	23	<0.001
Repeat no-go accuracy	62	26	56	27	0.30	29	18	60	22	<0.001
Color no-go accuracy	69	28	43	29	<0.001	27	22	49	28	0.009
Error awareness (% aware errors)	66	25	64	26	0.67	29	16	76	19	<0.001
Repeat error awareness	58	23	57	29	0.97	59	18	72	21	<0.001
Color error awareness	39	22	69	28	<0.001	31	18	80	20	0.002
Go RT (ms)	545.1	87.2	521.5	101.1	0.03	549.7	87.9	507.7	101.8	<0.001
Error RT (ms)	558.6	96.2	524.9	116.3	0.03	548.0	101.6	515.9	109.9	<0.001
Aware error RT	565.9	105.5	527.8	118.5	0.05	552.1	103.4	511.8	109.5	<0.001
Unaware error RT	550.5	103.1	514.9	110.2	0.09	542.6	107.3	527.5	120.1	<0.001
Error awareness RT (ms)	453.6	91.7	424.5	100.4	0.01	444.1	80.6	407.9	92.0	<0.001

*Accuracy (percentage of correct responses) data presented in table are not arcsine transformed and represent the observed overall accuracy rate and percentages of errors a participant was aware of. P-values represent paired-samples t-tests comparing early and late accuracy and error rates, as well as RTs within groups*.

#### Color Lure No-Go Error Awareness

Robust 2-Group × 2-Time (e.g., early, late) ANOVA for early and late EAT performance showed a significant main effect of time for awareness of color no-go errors, *T*_WJt_/c(1,37.2) = 108.38, *p* < 0.001, indicating that both the M/S TBI and control groups improved their awareness of color no-go errors. There was no main effect of group, *T*_WJt_/c(1,32.3) = 0.31, *p* = 0.57. The Group × Time interaction was also not statistically significant, *T*_WJt_/c(1,37.2) = 3.06, *p* = 0.09, for color no-go awareness. Both groups improved awareness of color no-go errors over the course of the EAT.

#### Color Lure No-Go Accuracy

When comparing the M/S TBI and control groups on early and late color no-go accuracy percentage there was no significant main effect of time, *T*_WJt_/c(1,54.6) = 0.62, *p* = 0.43. However, there was a significant main effect of group, *T*_WJt_/c(1,24.4) = 11.56, *p* = 0.003, and a significant Group × Time interaction, *T*_WJt_/c(1,54.6) = 30.85, *p* < 0.001. Decomposition of the interaction shows that M/S TBI performance on color no-go trials decreased from early to late, *T*_WJt_/c(1,20.0) = 31.10, *p* < 0.001, while control’s performance increased, *T*_WJt_/c(1,35.0) = 8.40, *p* = 0.007. Interestingly, the M/S TBI group had a significantly elevated first-half accuracy percentage, *T*_WJt_/c(1,33.2) = 37.25, *p* < 0.001, compared to controls, but no difference during the later half of the task on color no-go trials, *T*_WJt_/c(1,41.1) = 0.33, *p* = 0.57.

#### Repeat Lure No-Go Awareness

Comparisons of repeat no-go awareness for early and late EAT performance indicated a non-significant main effect of time for awareness of repeat no-go errors, *T*_WJt_/c(1,33.6) = 3.28, *p* = 0.08. There was no main effect of group, *T*_WJt_/c(1,27.9) = 1.79, *p* = 0.19, or Group × Time interaction, *T*_WJt_/c(1,33.6) = 3.07, *p* = 0.09 for repeat no-go awareness.

#### Repeat Lure No-Go Accuracy

There was a significant main effect of time, *T*_WJt_/c(1,46.4) = 9.20, *p* = 0.003, a significant main effect of group, *T*_WJt_/c(1,27.2) = 7.48, *p* = 0.02, and a significant Group × Time interaction, *T*_WJt_/c(1,46.4) = 21.04, *p* < 0.001. Decomposition of the interaction shows an initially worse repeat trial accuracy during the early half of the task for controls, *T*_WJt_/c(1,33.2) = 23.89, *p* < 0.001, followed by an improvement in repeat no-go accuracy by controls during the later half, *T*_WJt_/c(1,35.0) = 33.86, *p* < 0.001, that accounts for the interaction. The M/S TBI group showed no difference between repeat no-go accuracy early and late performance when compared to controls (*p*s > 0.32).

Comparisons of early and late performance for RTs indicated a significant main effect of time for go trials, *T*_WJt_/c(1,39.4) = 23.00, *p* < 0.001, error trials, *T*_WJt_/c(1,38.5) = 16.16, *p* < 0.001, awareness response trials, *T*_WJt_/c(1,41.6) = 23.75, *p* < 0.001, and aware and unaware errors, *T*_WJt_/c(1,31.7) = 13.98, *p* < 0.001 and *T*_WJt_/c(1,40.6) = 7.35, *p* = 0.01, respectively with both groups showing a decrease in RTs over time. There were no significant main effects of group or Group × Time interactions for RTs (*T*_WJt_/cs < 1.38, *p*s > 0.24).

.

### Change Score Analyses

Means and SDs for change scores as a function of group are included in **Table 5**. Significant mean differences were found between the control and M/S TBI groups on percent change from first half to second half performance for combined no-go trial error awareness, *t*(1,59) = 1.26*, p* < 0.001. Control participants showed improvement, whereas TBI participants either showed no improved or some decline. When separated by no-go trial type significant differences between groups was not present, *t*s < 1.66, *p*s > 0.06. No significant differences were found between groups for change scores of combined no-go trial accuracy, *t*(1,38.81) = 0.47*, p* = 0.64. However, when broken down by no-go lure type significant differences between the two groups was found for change scores for both color no-go accuracy, *t*(1,55) = 3.60*, p* = 0.001, and for repeat no-go accuracy, *t*(1,56) = 3.29*, p* = 0.002.

**Table 5:** Descriptive data and early to late behavioral change scores during the EAT as a function of group.

	M/S TBI (*n* = 24)		Control (*n* = 37)	
	Mean	SD	Mean	SD
No-go accuracy (% change)	3	40	7	29
Repeat no-go accuracy	5	66	71	82
Color no-go accuracy	-27	39	75	135
Error awareness (%change of aware errors)	-3	28	122	101
Repeat error awareness	3	50	26	43
Color error awareness	80	1.00	188	299

*Mean change scores represent the percentage of change from early to late performance on the EAT*.

### Correlations with Injury Severity

Duration of PTA correlated significantly with overall error awareness (*r* = -0.52, *p* = 0.009), repeat lure error awareness (*r* = -0.68, *p* < 0.001), and repeat lure no-go accuracy (*r* = -0.51, *p* = 0.01). The same pattern of significant correlation carried over into analyses comparing early to late behavioral performance (early task correlations; *r*s < -0.51, *p* < 0.01 and late task correlations *r*s < -0.42, *p* < 0.02). This pattern of correlation suggests that M/S TBI participants with higher duration of PTA had lower error awareness and accuracy, specifically with repeat lure no-go trials. There were no significant correlations with LOC duration (all *p* > 0.05).

### Event-Related Potential Analyses

**FIGURE 2:**
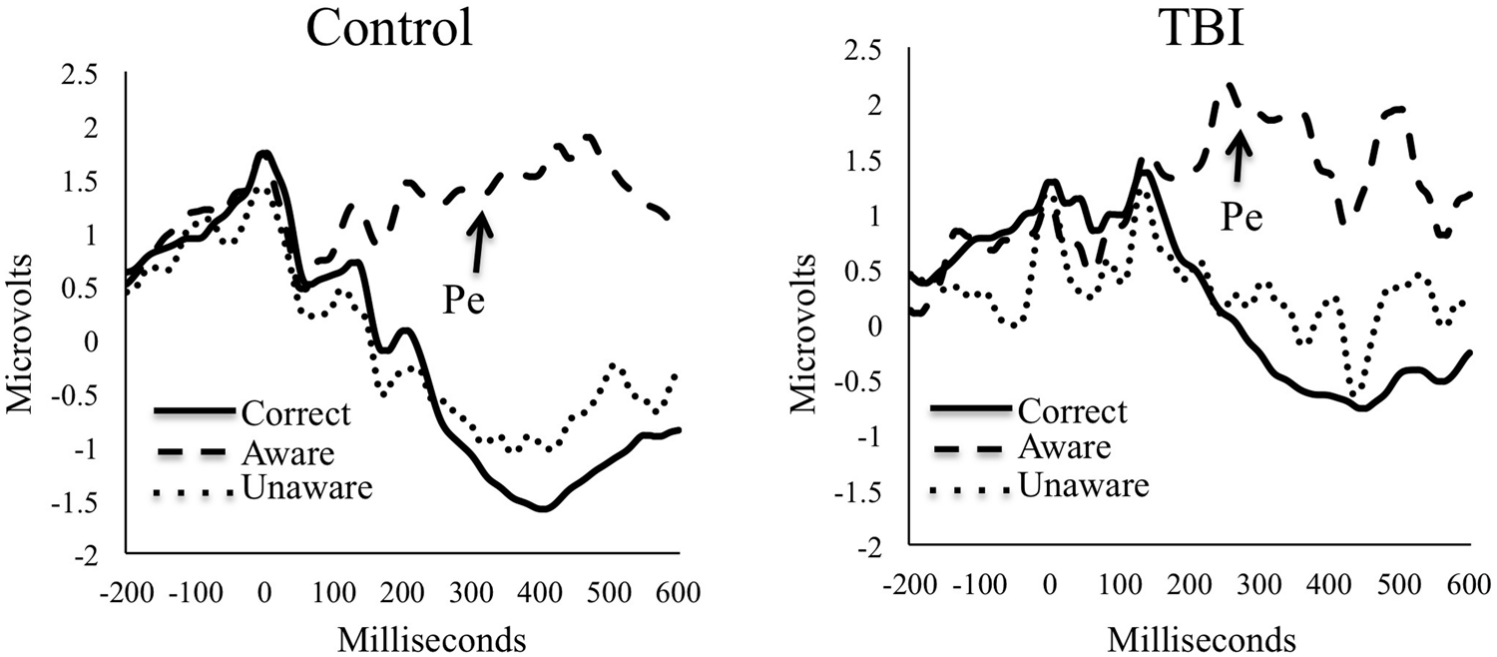
**Grand average waveforms for the Pe component by group**.

The Pe ERPs for both groups are presented in **Figure 2**. Groups did not significantly differ on numbers of trials used for ERP analyses for any condition (*p*s > 0.05). The M/S TBI group had an average (SD) of 45.4 (21.1) aware errors, 32.4 (25.4) unaware errors, and 413.5 (85.3) correct trials. Controls had means (SD) of 60.9 (25.5) aware errors, 24.5 (18.9) unaware errors, and 805.3 (61.8) correct trials. For the TBI participants, mean (SD) Pe amplitude for unaware errors was 0.1 (1.3) microvolts with a range from -2.0 to 3.7. For aware errors the mean amplitude was 1.9 (2.2) with a range of -2.0 to 5.7. For the control participants, mean (SD) Pe amplitude for unaware errors was -0.2 (2.2) with a range of -8.7 to 6.2 microvolts. For aware errors, the mean Pe amplitude was 1.7 (2.4) with a range of -6.4 to 6.3.

As is clear from the amplitude means for the Pe, there was a significant main effect of awareness, *T*_WJt_/c(1,29.0) = 28.78, *p* < 0.001, showing that the awareness of errors corresponded with increased Pe amplitude for both groups. There was no significant main effect of group, *T*_WJt_/c(1,37.9) = 0.59, *p* = 0.45, or Group × Awareness interaction, *T*_WJt_/c(1,29.0) = 0.15, *p* = 0.70, when comparing M/S TBI with controls on Pe amplitudes.

## Discussion

Findings indicate that M/S TBI and control groups were similar on ERP (i.e., Pe amplitude) indicators of error awareness. Similarly, analyses of RT and accuracy data suggest similarities between groups in accuracy and awareness. A different picture emerges, however, when data are split into first- and second-halves to examine attention and task learning effects. Whereas both groups became faster over the course of the task, TBI participant accuracy decreased or stayed the same over time, whereas the control group improved performance over time completing the task—both for color and repeat lure types. Importantly, overall error awareness and percent change improved over time for the control participants. Participants with M/S TBI, however, remained similar in awareness across the first and second halves of the task for repeat lures, but did show some improvements for color lures. The color lures are notably easier as participants only need to identify congruent trials. Thus, on the more difficult repeat lures, the individuals with M/S TBI were unable to improve error awareness during the course of the task.

The results of differential amounts of improvement on the EAT as a function of group and time on the task are mixed relative to previous work in the area of error awareness in a M/S TBI sample. Similar to previous studies, results from the current study show improvements in RTs across groups over time (Larson et al., 2007, 2009; Larson and Perlstein, 2009); however, current results showed significant accuracy differences between groups over time. Previous studies did not look at differential task performance in the early and late halves of their respective tasks. Current findings show no differences between groups when looking at the entire EAT, but analyses comparing early and late performance between groups show that M/S TBI participants failed to improve awareness and accuracy performance compared to control participants.

One possible hypothesis is that the differences in awareness between groups on difficult repeat lure trials over time are most likely related to difficulties with sustaining attention that can adversely effect emergent and anticipatory awareness (Crosson et al., 1989). The M/S TBI group did not attend well enough to the task to stop them from making errors on similar types of trials as they were happening. However, easier color trials were attended to in a manner that performance improved during the EAT task. Another possible interpretation is that the participants with M/S TBI required increased cognitive effort to maintain awareness and, after expending considerable effort, showed decreased accuracy over time. A third possibility that should be strongly considered is regression to the mean and sampling error. Specifically, control participants performed worse on the first half of the task then improved to the point of near-equivalence of those with TBI on the second half of the task. Percent change scores show improvement in the controls relative to those with TBI, but it is indeed possible that these difference only reflect regression to the mean and nothing further. It is also possible that sampling error and variability contribute to the current findings.

Given these findings, there are three key follow-up research possibilities: (1) declines in complex attentional processes may lead to results showing differential patterns in accuracy relative to control participants, (2) attentional difficulties may be associated with decreased levels of error awareness over time for the M/S TBI group despite similar physiological responses as controls to errors, (3) sampling error and regression to the mean may have influenced findings and replication is needed. From an attention perspective, in a previous study no-go errors (false positive button presses) during the Sustained Attention and Response Task (a task similar to the EAT, but with a focus on sustained attention over time) were associated with impaired error awareness, suggesting that lapses in sustained attention or inhibition may result in greater numbers of unaware errors (McAvinue et al., 2005). Executive control difficulties can be seen through poor sustained attention, inhibition of prepotent responses, and monitoring of environmental changes. Attention, inhibition, and monitoring all draw upon resources from the environment and/or require endogenous behavioral control to maintain a goal-directed focus, which can be compromised following a TBI and lead to increased attentional lapses and decreased awareness of errors (O’Keeffe et al., 2007). O’Keeffe et al. (2007) proposed that more cognitively simple tasks will increase the challenge of maintaining attention and alertness to combat the monotony of the task, but more cognitively challenging tasks will be more stimulating and increase alertness to task demands.

The EAT task requires sustained attention and is cognitively demanding throughout its duration. Participants must learn and remember two competing rules and various instructions related to the signaling of an aware error. Results show that participants were able to quickly master those rules and procedures without difficulty, as evidenced by the fact that all but one severe TBI participant were able to learn the task requirements on the first practice session. Due to the length of the task and the speed at which stimuli are presented the cognitive demands do not reduce but may wane once participants are engaged in the task due to monotony and fatigue. There is some automation of responses with the majority of trials being go-trials, potentially resulting in difficulty maintaining attention and vigilance to performance. The current characterization of decreased awareness of errors due to attentional drift is consistent with other studies in M/S TBI survivors who show poor sustained attention over time that is related to overall awareness (O’Keeffe et al., 2004, 2007; McAvinue et al., 2005; Dockree and Roberston, 2011).

For the ERPs, the Pe component showed significant differences for awareness demonstrating increased amplitude Pe amplitude for aware compared to unaware errors—consistent with previous findings (O’Connell et al., 2007; Charles et al., 2013). Contrary to our hypothesis, the findings from the Pe component, representing conscious error awareness, showed no significant differences between the M/S TBI and control groups. A lack of group differences on the Pe would seem to indicate that the Pe is intact in those with M/S TBI and signals conscious awareness of errors. In other words, similar Pe amplitudes between the M/S TBI group and controls indicates that both groups had similar electrophysiological representations of conscious error awareness. There is some debate about whether or not the Pe is a binary indicator of error awareness or if it corresponds to error awareness inputs from other sources such as the error-related negativity (Overbeek et al., 2005; Steinhauser and Yeung, 2010; Shalgi and Deouell, 2013).

Elevated Pe amplitude is thought to represent awareness of errors with amplitudes of unaware errors being similar to correct responses (Hughes and Yeung, 2011), as is the case in this study. Analogous to current findings, the Pe component did not differentiate between TBI and controls in other studies of individuals with M/S TBI not related to conscious error awareness (Larson et al., 2007, 2009). However, previous results also show that levels of awareness drawn from differences in self-reported and significant other-reported awareness are positively correlated with Pe amplitudes (Larson and Perlstein, 2009). Further work is needed to confirm if awareness of day-to-day performance difficulties correlates with conscious awareness of errors in real time evaluation of the Pe component of the ERP. Data to date, however, indicate that Pe amplitudes do not differ between those with TBI and healthy controls. Indeed, studies of mild TBI also do not show differences in Pe amplitude relative to controls (Pontifex et al., 2009; Larson et al., 2012).

Current findings support the possibility that individuals with M/S TBI have difficulty improving performance and awareness over time. Such difficulties could be manifest during learning tasks or, perhaps, even during classroom or workplace activities where uninjured individuals are continuing to progress and the individuals with M/S TBI plateau. Fatigue, difficulties with sustained attention, and high cognitive effort to ensure accurate performance all likely play a role in these difficulties. Similar patterns may emerge in long neuropsychological testing sessions or on repeated recall tests where participants are required to sustain attention, increase accuracy, and improve performance over time, although future research is clearly needed to test these possible real-world implications of the current results.

## Limitations

One important limitation of this study is the nature of the sample itself. Specifically, a high degree of heterogeneity in the TBI sample may account for the high functioning cognitive abilities. Notably, the participants were mostly chronic and high functioning (i.e., able to respond to flyers and advertisements and complete a relatively difficult task). One TBI participant in particular scored very well on all neuropsychological and test measures driving up the mean scores (see the high scores and the range in **Table 2**). There were also significant correlations with length of PTA and EAT behavioral performance. Thus, having more participants with more severe TBI as defined by PTA may have led to more differences between TBI and control participants. We also note that there was a wide range of injury severity. We gathered injury severity data from medical records in as many cases as possible. We note that while medical records do provide additional confirmation of severity there are potential confounds in using indices such as GCS and LOC for severity classification due to medical procedures such as incubation, induced coma, and surgery (Lezak et al., 2012). That said, we used validated interviewing techniques for determining injury severity and length of PTA. Future studies with a more homogeneous acute TBI sample are needed to further elucidate the role of attention in the complex process of error awareness. Similarly, control and TBI participant groups differed on several important demographic variables including age, and depression and apathy severity. Notably, participants with TBI did not show clinically elevated levels of depression or apathy, but the differences in age and emotional functioning relative to the control group could have contributed to the ability to sustain attention and error awareness over time. Another potential limitation of the current study was the exploratory use of the EAT task with a M/S TBI sample. There has been no previous use of this type of task with this population and there will need to be replication in order to determine reliability of results. However, the EAT has been successfully used in fMRI studies with chronic substance abuse populations (Hester et al., 2007, 2009), and in healthy controls for previous ERP studies with similar results across studies (O’Connell et al., 2007; Orr and Hester, 2012). Finally, we did not have sufficient useable error trials to separate the Pe amplitude data by early and late task performance. It is possible the Pe differed as a function of time on task, but we were not able to test this possibility due to the potential for unreliable ERP waveforms without adequate signal-to-noise ratio.

## Conclusion

We found that individuals with M/S TBI have a different process of error awareness using behavioral measures than healthy controls. Individuals with M/S TBI did not improve their performance over time, while control participants improved their accuracy, but started lower than those with TBI. Awareness of errors improved for both groups in the color task, but those with M/S did not improve in recognition of repeat errors over time. Consistent with previous studies in TBI, there were no between-group differences for Pe amplitude. Results suggest subtle awareness difficulties relative to controls that are more pronounced over time and may be related to sustained attention. The current study provides support for continued exploration of performance and awareness across task duration and the effects of task requirements on behavioral and electrophysiological indicators of error awareness.

## Conflict of Interest Statement

The authors declare that the research was conducted in the absence of any commercial or financial relationships that could be construed as a potential conflict of interest.

## Footnotes

1 In order to address potential confounds due to age differences between the control and M/S TBI groups we completed additional analyses after removing the three oldest M/S TBI participants leaving *n* = 21 for the M/S TBI group. Removing these three participants resulted in an age match between the groups, *t*(1,30.27) = -1.92, *p* = 0.07. The mean (SD) age for the M/S TBI group with the three M/S TBI participants removed was 27.05 (8.26) years, and the controls had a mean age of 23.19 (5.50) years. There were no other differences in demographic or neuropsychological variables. Results indicated similar results with the exception of a significant main effect of time for repeat lure no-go awareness, *T_WJt_/c*(1.0,30.2) = 5.34, *p* = 0.03, and a significant interaction for color lure no-go awareness, *T_WJt_*/c(1.0,31.0) = 5.04, *p* = 0.03 with decomposition showing a greater level of improvement in awareness for the control group, *T_WJt_*/c(1.0,34.0) = 130.63, *p* < 0.001 over the M/S TBI group, *T_WJt_*/c(1.0,17.0) = 21.34, *p* = 0.001, although both groups still improved awareness over time. The results from this separate analysis do not change the pattern or interpretation of results of the main analyses completed for this study.
